# The South African Flag Sign: A Key Indicator of Acute D1 Occlusion

**DOI:** 10.7759/cureus.81468

**Published:** 2025-03-30

**Authors:** Johannes Luckmann, Soe Win, Temitope Ajagbe, Thisara Samarawickrama

**Affiliations:** 1 Cardiology, Univeristy of Exeter Medical School, Truro, GBR; 2 Cardiology, Royal Cornwall Hospital, Truro, GBR; 3 General Medicine, Royal Cornwall Hospital, Truro, GBR

**Keywords:** acute coronary syndrome, d1 occlusion, ecg patterns, interventional cardiologist, south african flag sign

## Abstract

The South African flag sign (SAFS) on an ECG suggests an occlusion in the first diagonal branch of the left anterior descending (LAD) coronary artery, particularly in the context of acute chest pain. Due to its unique findings, it is often overlooked or misidentified. We present four cases in which this finding was identified on ECG, along with the corresponding angiographic images and clinical details. Two of these had an acute diagonal branch occlusion, which was treated with stent insertion. The other two cases had some angiographic changes but had no stent inserted. All of these cases showed a varying degree of left ventricular dysfunction and troponin rise, highlighting the importance of recognition and prompt management. On subsequent cardiac MRI, one patient was determined to have an aborted infarct, and another was suspected of the same. The urgency with which the SAFS should be expedited to invasive imaging should match that of other acute signs of occlusion, such as the new left bundle branch block (LBBB) in the context of chest pain. Increased awareness of the SAFS can prevent these cases from being overlooked and improve patient outcomes by ensuring they receive timely care.

## Introduction

Electrocardiograms (ECG) are a crucial investigation in the diagnosis of acute coronary syndrome (ACS), guiding rapid intervention in coronary artery occlusions. Among various ECG patterns, which indicate severe myocardial ischemia, the South African Flag sign (SAFS) is seen in occlusions of the first diagonal branch (D1) of the left anterior descending (LAD) coronary artery. On classic 12 lead ECGs, the SAFS characteristically exhibits pronounced ST-segment elevation in leads I, aVL, and V2, and ST-segment depression in lead III. These changes thus resemble the green band in the South African flag. The isolated rise in V2 is due to ischemia in the upper septal area, which D1 typically supplies. A lack of awareness of these non-contiguous ST changes causes these findings to often be disregarded, misinterpreted for lateral infarcts due to ST changes in leads I and aVL, or missed entirely. In this case series, we present four cases of the classical SAFS on ECG, with each having varying findings and outcomes. These cases highlight the importance of awareness of the SAFS on ECG, as well as emphasizing the urgency with which these cases should be dealt with. This is because they can suggest a complete coronary occlusion, requiring prompt escalation, despite being classified as an NSTEMI.

## Case presentation

Patient 1

**Figure 1:**
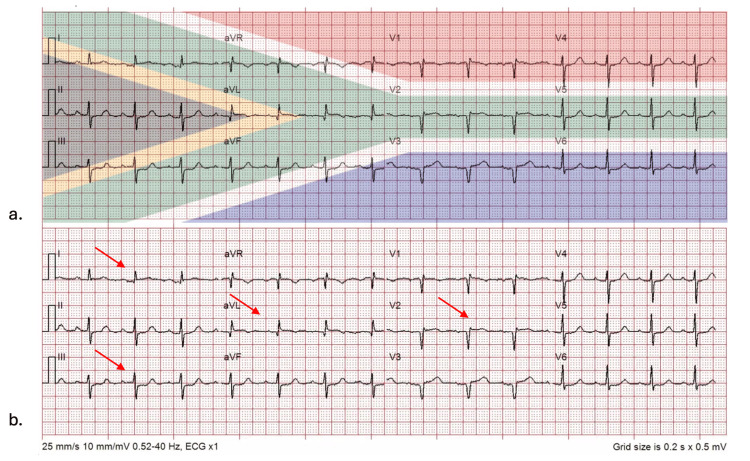
a) ECG of Patient 1 superimposed with the South African flag. b) The same ECG showing ST elevation in leads I, aVL, and V2, and ST depression in lead III. These changes align with the green band of the South African flag, providing a classic yet subtle example of SAFS. SAFS, South African flag syndrome; ECG, electrocardiogram

A 64-year-old female, with a history of hypertension, was admitted due to extremely severe central chest pain. She received glyceryl trinitrate (GTN) spray, aspirin, and ticagrelor, as well as blood tests, and an ECG was taken. ECG showed 1 mm ST elevation in leads I, aVL, and V2, with 1 mm ST depression in lead III (Figure 1). Apart from appearing extremely anxious, there were no examination findings of note. Initial troponin levels were raised at 1,095 ng/dL, rising further to 1,613 ng/dL. In the context of acute chest pain, these specific ECG changes, along with the elevated troponin levels, raised a high suspicion of a D1 occlusion.

**Figure 2:**
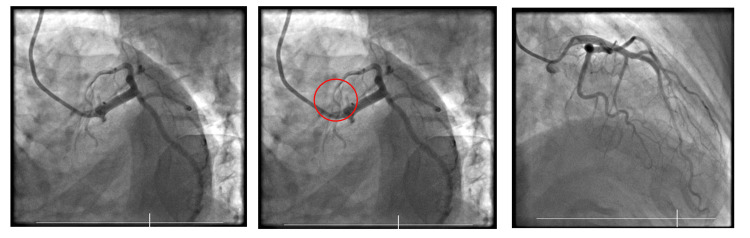
ICA images of Patient 1, showing 40-50% obstruction in D1. ICA, invasive coronary angiogram

An invasive coronary angiogram (ICA) revealed that the left main stem (LMS), LAD, left circumflex (LCx), and right coronary artery (RCA) had TIMI grade 3 flow. D1 had 40-50% eccentric stenosis at the ostium, seen only in caudal images (Figure 2).

A subsequent ECHO showed a reduced left ventricular ejection fraction (LVEF) of 36-40% with a globular and markedly hypokinetic apex. A cardiac MRI revealed no evidence of Takotsubo cardiomyopathy nor any late gadolinium enhancement. It did show, however, increased edema in the midanteroseptal and anterior wall implying an aborted infarct.

This patient was hereafter managed as MINOCA, and she was commenced on Aspirin 75 mg OD, Clopidogrel 75 mg OD, and Atorvastatin 80 mg OD as part of standard secondary prevention, along with Lansoprazole 15 mg OD. She was newly prescribed Bisoprolol 1.25 mg BD, Dapagliflozin 10 mg OD, and Sacubitril/Valsartan 24/26 mg BD for heart failure with reduced ejection fraction. Eplerenone was withheld due to low systolic blood pressure. The patient was not diabetic.

Patient 2

**Figure 3:**
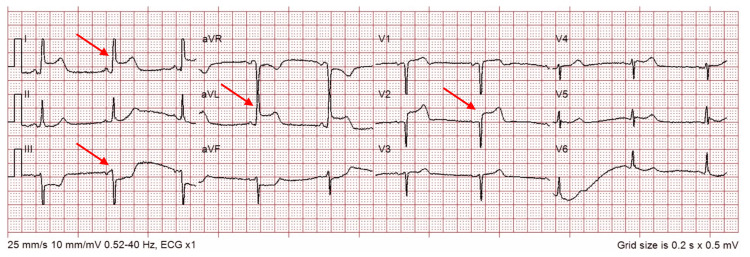
ECG of Patient 2 showing ST elevation in leads I, aVL, and V2, along with ST depression in lead III, highlighted by the arrows, the SAFS. SAFS, South African flag sign; ECG, electrocardiogram

A 61-year-old female, with a history of hypertension and GORD, was brought in by ambulance due to severe burning chest pain at rest lasting approximately three minutes. This pain had self-resolved when the ambulance crew arrived. Upon admission, she developed severe chest pain once again. On examination, the patient was alert and tachycardic, with no other findings of note. An ECG showed ST elevation in leads I, aVL, and V2, along with ST depression in lead III, the classical SAFS (Figure 3). Troponins were raised at 4,842 ng/dL. Given the ECG and laboratory findings, the initial burning pain, which could be misinterpreted as non-cardiac, was considered as ischemic.

**Figure 4:**
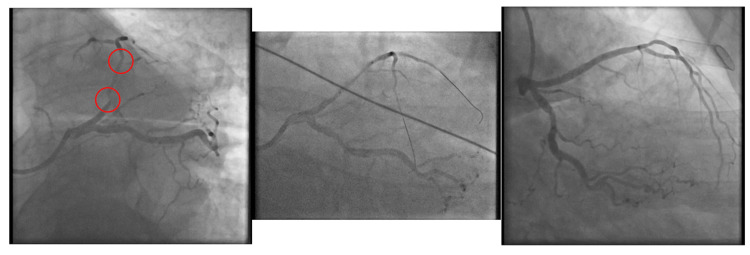
ICA images of Patient 2 revealing severe disease in the LAD and D1, as well as an image post-stent insertion. The occlusion in this case was diagnosed through the identification of the SAFS, highlighting its diagnostic potential. ICA, invasive coronary angiogram; SAFS, South African flag sign

Consent for an ICA was gained and revealed an unobstructed LMS, mild disease in the LCx, and dominant RCA, with moderate disease. The LAD showed severe proximal disease extending to the D1 bifurcation with 50% eccentric stenosis thereafter, with severe disease at the ostium of D1 (Figure 4).

She subsequently had primary PCI to LAD. After a successful post-PCI result, it was decided that she would have a staged procedure with a pressure wire study +/- PCI to RCA, which was done six days after the primary PCI procedure. The fractional flow reserve was 0.89 after the adenosine steady-state with no drift. Therefore, the RCA lesion was not functionally significant and did not require stenting; instead, it was medically managed.

An ECHO showed an akinetic left ventricular apex, apical septum, and lateral segments, with hypokinetic mid-lateral and mid-anterior septum segments. This was consistent with the PCI territory. Furthermore, the LVEF was reduced by 45-49%. She also had a dilated left atrium and mild tricuspid, aortic, and mitral regurgitation.

A lipid profile revealed a total cholesterol of 10.5 mmol/L and triglycerides of 4.7 mmol/L. She was discharged on the following medications one week after admission: Aspirin 75 mg OD, Ticagrelor 90 mg BD, Bisoprolol 2.5 mg OD, Lansoprazole 15 mg OD, Losartan 25 mg OD, Ezetimibe 10 mg OD, as well as GTN sublingual spray PRN for anginal relief. The patient was allergic to statins, therefore a follow-up appointment with the lipid specialist was booked and Fenofibrate 160 mg was prescribed. DAPT was continued for 12 months, followed by aspirin indefinitely. Cardiac rehabilitation in the community was also offered. 

Patient 3

**Figure 5:**
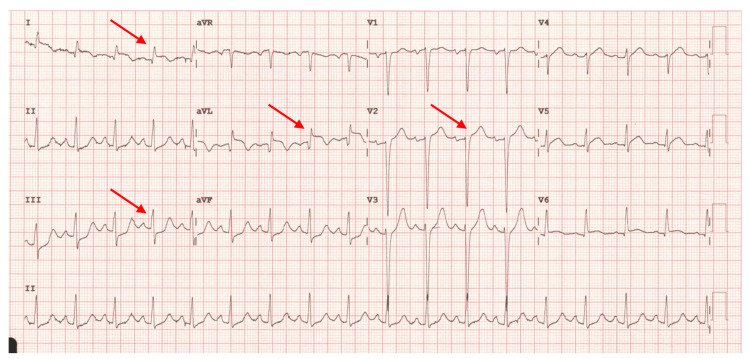
ECG of Patient 3 showing ST elevation in leads I, aVL, and V2, along with ST depression in lead III, highlighted by the arrows, the SAFS. SAFS, South African flag sign; ECG, electrocardiogram

A 65-year-old male, with a 30-pack-year smoking history, presented to his GP after developing chest pain at rest. A subsequent ECG showed ST elevation in leads I, aVL, and V2, along with ST depression in lead III (Figure 5). His troponins were raised at 35,801 ng/dL. Given the patient's smoking history, ECG findings, and raised troponin, a coronary artery occlusion, likely D1, was considered the most likely explanation.

**Figure 6:**
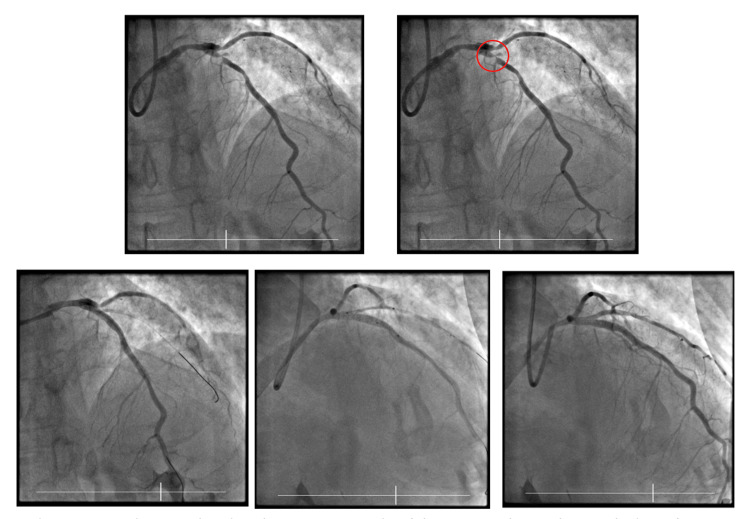
ICA images showing severe stenosis of the LAD and complete occlusion of D1 in the context of SAFS. Subsequent images demonstrate stent insertion into the aforementioned vessels. ICA, invasive coronary angiogram; SAFS, South African flag sign; LAD, left anterior descending

Urgent ICA revealed 30% stenosed LMS, with 99% stenosis of the LAD at the D1 level, with the D1 branch being 100% blocked proximally. The LCx had at least moderate disease in the proximal vessel, and the RCA showed mild proximal, and moderate to severe disease in the mid vessel (Figure 6).

Following this, PCI was performed on the LAD and D1. An excellent post-PCI result with TIMI 3 flow in LAD and D1 was achieved. An inpatient ECHO thereafter showed a reduced LVEF of 40-45%. The left ventricle had an akinetic anterior, apical lateral, mid inferolateral, and anterolateral walls. 

The patient was discharged seven days later with the following medications: Aspirin 75 mg OD, Atorvastatin 80 mg OD, Bisoprolol 1.25 mg BD, Dapagliflozin 10 mg OD, Ramipril 2.5 mg OD, Ticagrelor 90 mg BD, and GTN sublingual spray to use PRN in the event of chest pain. 

Following this, there was an MDT discussion with the Derriford cardiothoracic department about suitability for CABG, due to the other diseased vessels discovered on ICA. Unfortunately following the PCI, he developed rectal cancer, and due to the corresponding treatment, the consideration for CABG was put on hold to allow for the rectal tumor to be resected. Following this, a pressure wire study was performed on the LAD, which showed the functional flow decrease from 0.94 to 0.81 after adenosine infusion with no drift, a borderline negative result. A cardiac MRI revealed only mild inducible ischemia, and therefore, this decision was made for this patient to be managed medically. The patient has remained completely free of chest pain since the PCI to the LAD.

Patient 4

**Figure 7:**
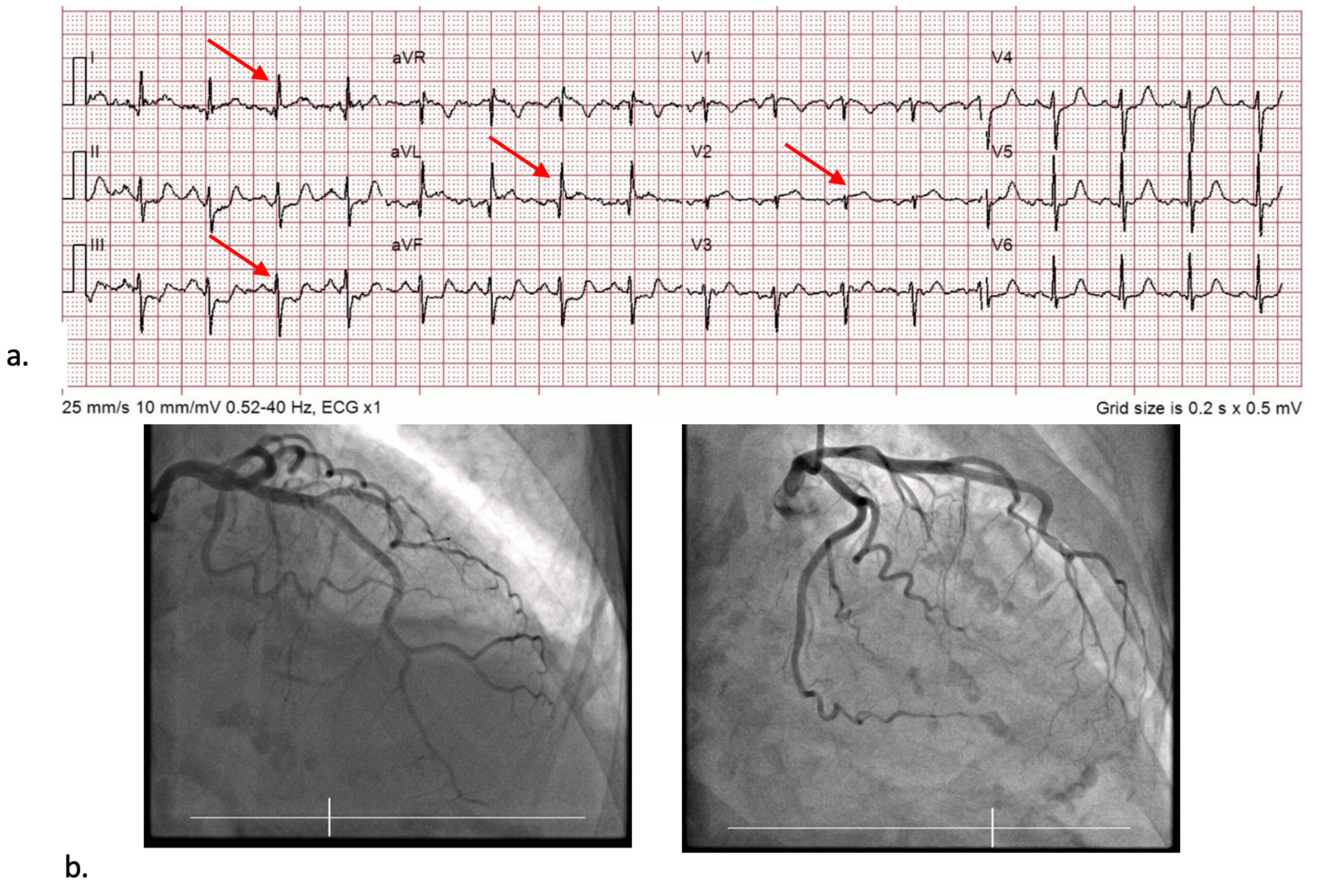
a) ECG of Patient 4 showing ST elevation in leads I, aVL, and V2, along with ST depression in lead III, highlighted by the arrows, the SAFS. b) ICA images show unimpaired D1 flow, highlighting the low specificity of the SAFS, as not all who exhibit the sign have acute occlusions in D1. ICA, invasive coronary angiogram; SAFS, South African flag sign; ECG, electrocardiograms

An 87-year-old female, previously treated for a pulmonary embolism three months prior, called for emergency services due to acute band-like chest pain, radiating to her back, lasting several hours. On examination, she was alert and oriented and slightly tachycardic, with nil else of note. An ECG taken by paramedics was interpreted as a posterior or anterolateral STEMI, therefore aspirin 300 mg and GTN spray were given. The ECG showed ST elevation in leads I, aVL, and V2, with ST depression in lead III, another classical SAFS ECG (Figure 7a). No further ECGs were performed with leads V7-V9 to rule out a posterior STEMI. The initial troponin level was 781 ng/dL, which later rose to 4,823 ng/dL. She was expedited directly to the catheter laboratory for primary percutaneous coronary intervention (PPCI). After being consented to the procedure, the ICA revealed all coronaries to be unobstructed, including D1 (Figure 7b).

Due to a pulmonary embolism in the preceding months and the finding of normal coronaries, she underwent a further CTPA scan, which revealed no acute embolism. A subsequent ECHO revealed an estimated reduced LVEF of 40-45%, as well as regional wall motion abnormalities in the mid to apical regions, suggestive of Takotsubo cardiomyopathy, which she was subsequently diagnosed with. Unfortunately, a cardiac MRI could not be performed as her renal function was too impaired. No further imaging was considered. However, given the severe presenting chest pain, significant troponin rise, and subsequent resolution of ST changes on the ECG, we believe it is likely that this, too, was an aborted infarct.

She was discharged two days later to the care of her GP and for follow-up with community heart failure nurses. The following medications were prescribed: Dapagliflozin 10 mg OD, Sacubitril/Valsartan 24/26 mg BD, Spironolactone 25 mg OD, and Edoxaban 30 mg OD for continuing pulmonary embolism prophylaxis. It was decided the benefits of co-prescribing antiplatelets alongside a DOAC outweighed the risks. 

## Discussion

The role of ECGs in the identification and management of ACS cannot be overemphasized. It is a quick, easy-to-use investigation that helps guide clinicians to manage patients through the appropriate pathways [1]. Furthermore, ECG findings can guide interventionists to the culprit artery, accelerating the time to reperfusion and improving patient outcomes.

The SAFS, first coined by Laszlo Littman in 2015, is a rare but crucial ECG finding that requires prompt recognition and intervention [2,3]. It is characterized by ST elevation in leads I, aVL, and V2, with ST depression in lead III [2-4]. These non-contiguous ST changes, often misdiagnosed as lateral or inferior MI, pose a diagnostic challenge to clinicians, sometimes even being missed entirely [5,6]. As seen in case 1, the ST changes associated with the SAFS can be very subtle, despite complete coronary occlusion. Studies have shown that this pattern indicates a critical or complete occlusion of D1 of the LAD [2-4]. The significance of this lies in the proportion of left ventricular myocardium at risk, which varies between patients depending on the size of the vessel. Those who supply more than 10% of myocardium through D1 require prompt PCI to minimize myocardial necrosis [7,8]. 

The isolated ST elevation in V2 can frequently confuse interpreters as it does not correspond to the relevant arterial territory of either a lateral or high lateral STEMI [4]. The significance of this lies in the fact that this is not an MI caused by an occlusion of the LCx, and therefore involving the lateral wall, for which it can be often confused. This can then misguide the interventional cardiologist by leading them to look for an occlusion in the LCx rather than D1 [9]. A high or very proximal D1 may supply part of the upper septum or upper anterior wall, which is represented by V2 [4]. Furthermore, the septal perforator artery (SP1), which often arises from the same region, may also have concurrent disease [10]. Concurrent disease in these vessels, both of which supply the septum, can worsen septal hypokinesis, worsening left ventricular function. A thrombus in the LAD-D1-SP1 region can give a non-contiguous but still life-threatening or debilitating outcome, which is why it should not be missed [11].

Current practice categorizes myocardial infarcts based on the ST segment, classifying them as either STEMI or NSTEMI. While this approach allows for rapid identification of critical occlusions through corresponding ST elevation, those patterns that do not exhibit traditional ST elevation, such as Wellens’ syndrome, De Winters T waves, and the SAFS, are overlooked. A meta-analysis of over 40,000 patients has shown that approximately 25% of NSTEMI diagnoses had complete coronary occlusions, underscoring the pitfalls of the current STEMI/NSTEMI guidance [9,12].

In contrast to this, the occlusive myocardial infarction (OMI) and non-occlusive myocardial infarction (NOMI) classification categorizes infarcts based on coronary status, rather than ST characteristics. Although STEMI falls within the OMI category, other patterns, which typically do not fall under the STEMI umbrella, such as SAFS, DeWinter’s, and Aslanger's patterns, do classify as OMI. This emphasizes the importance of urgent ICA and potential PPCI in all these patients, with cases of SAFS being managed as promptly as traditional STEMI patients are managed [8]. The 2023 European Society of Cardiology ACS guidelines support this, with level 1 class A evidence stating that patients with a high suspicion of acute coronary occlusion should receive emergency angiography as soon as possible, even if classified as NSTEMI [13].

As the presented cases have shown, with only half having significant stenosis of D1, the sensitivity and specificity for D1 occlusions in the presence of the SAFS are low, with the exact diagnostic accuracy remaining unclear. It is nevertheless a crucial finding, which should not be overlooked nor underestimated. It echoes the principles of acute left bundle branch block (LBBB) management. A new LBBB in the context of acute chest pain has historically been included in the STEMI criteria, but due to its low sensitivity, its role in urgent management has been debated. Only 15% of patients who exhibit a new LBBB have an acute occlusion; however, despite this, it is investigated and managed with the utmost severity, as acute occlusion should not be missed [14,15]. Similarly, when SAFS is detected on an ECG, the patient should be promptly expedited to ICA, as cases with occlusion should not be overlooked. The outcomes for each patient were variable, with reduced ejection fraction being a common complication. Further research is needed to establish the consequences of isolated D1 occlusions, which would allow for the stratification of care urgency.

## Conclusions

These cases have highlighted the importance of identifying the SAFS on ECG. Despite variability in D1 occlusion, recognition is essential in ACS triage. This is because the SAFS may indicate an acute coronary occlusion despite being classified as an NSTEMI, therefore warranting urgent ICA to avoid potential complications. Increased awareness and a high index of suspicion for SAFS should be maintained when patients present with atypical ECG changes, particularly in the presence of acute chest pain. This can ensure prompt recognition and timely intervention, improving patient outcomes. Further studies are needed to assess the diagnostic accuracy and optimal management strategies in patients who display the SAFS.
